# Papillocystic Variant of Acinar Cell Pancreatic Carcinoma

**DOI:** 10.1155/2010/242016

**Published:** 2010-03-03

**Authors:** Jasim Radhi, France Tse, Michael Marcaccio

**Affiliations:** ^1^Department of Pathology, McMaster University Medical Centre, Hamilton, ON, Canada L8N 3Z5; ^2^Department of Gastroentrology, McMaster University Medical Centre, Hamilton, ON, Canada L8N 3Z5; ^3^Department of Surgery, McMaster University Medical Centre, Hamilton, ON, Canada L8N 3Z5

## Abstract

Acinar cell pancreatic carcinoma is a rare solid malignant neoplasm. Recent review of the literature showed occasional cases with papillary or papillocystic growth patterns, ranging from 2 to 5 cm in diameter. We report a large 10 cm pancreatic tumor with papillocystic pathology features involving the pancreatic head. The growth pattern of these tumors could be mistaken for intraductal papillary mucinous tumors or other pancreatic cystic neoplasms.

## 1. Introduction

Acinar cell carcinoma of the pancreas is a rare tumor and account for less than 2% of all pancreatic carcinoma. These tumors are commonly large well-circumscribed solid and highly cellular lesions. Recent literature review showed occasional rare cases with papillary or papillocystic growth pattern that can be mistaken for mass forming cystic neoplasms of the pancreas [1]. Cystic pancreatic tumors represent a diverse collection of tumors with varied malignant potential and clinical presentation [2]. The general differential diagnosis includes intraductal papillary mucinous neoplasms (IPMNS), cystic neuroendocrine tumors, solid pseudopapillary tumor and mucinous cystic neoplasms. However, these tumors carry more indolent and protracted clinical course than acinar cell carcinoma [3]. The correlation of clinical, radiographic, histologic, and immunohistochemical findings would be helpful to establish the accurate diagnosis and management.

## 2. Case Presentation

**Figure 1:**
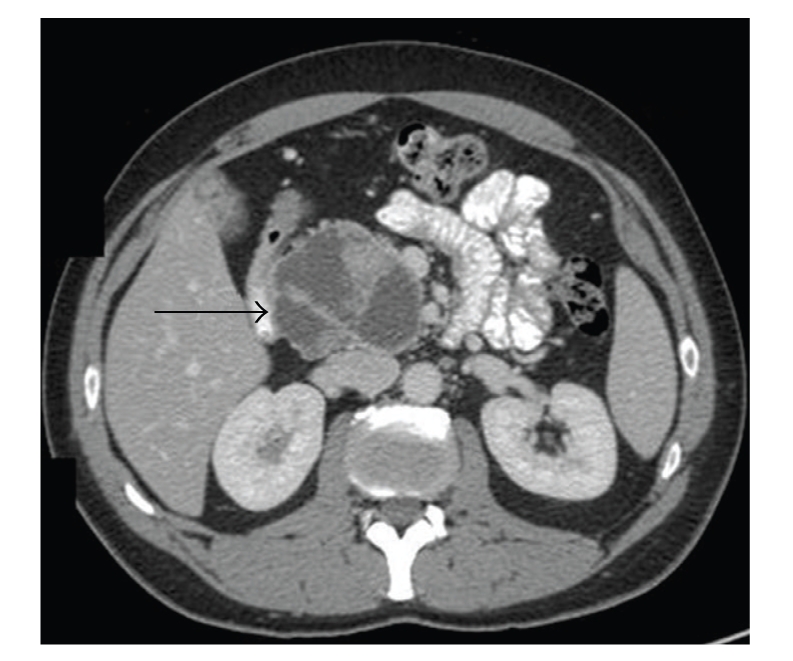
CT scan showed a large heterogeneous cystic lesion in the head of the pancreas (arrow).

**Figure 2:**
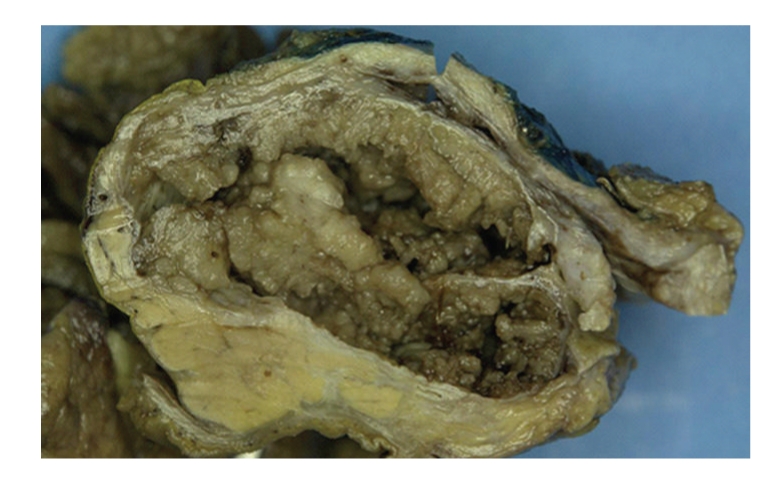
Gross appearance of a large papillocystic pancreatic lesion.

**Figure 3:**
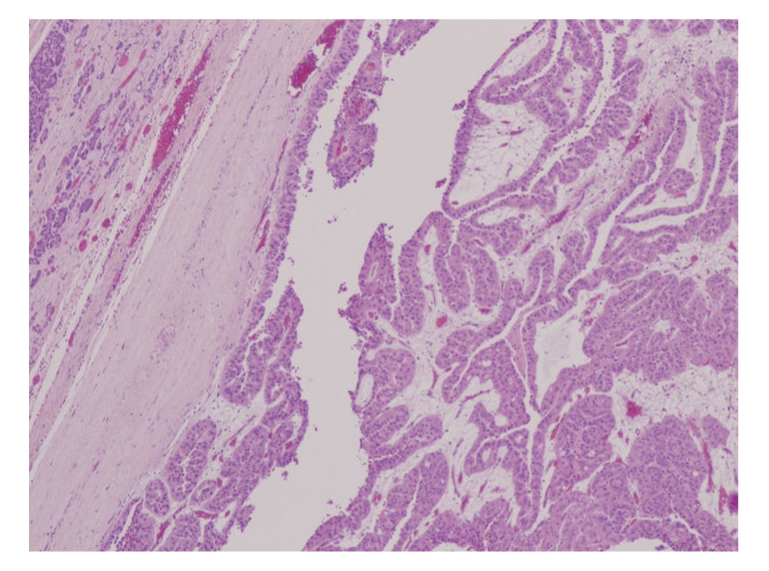
Photomicrograph showing papillary projections lined by cuboidal cells.

**Figure 4:**
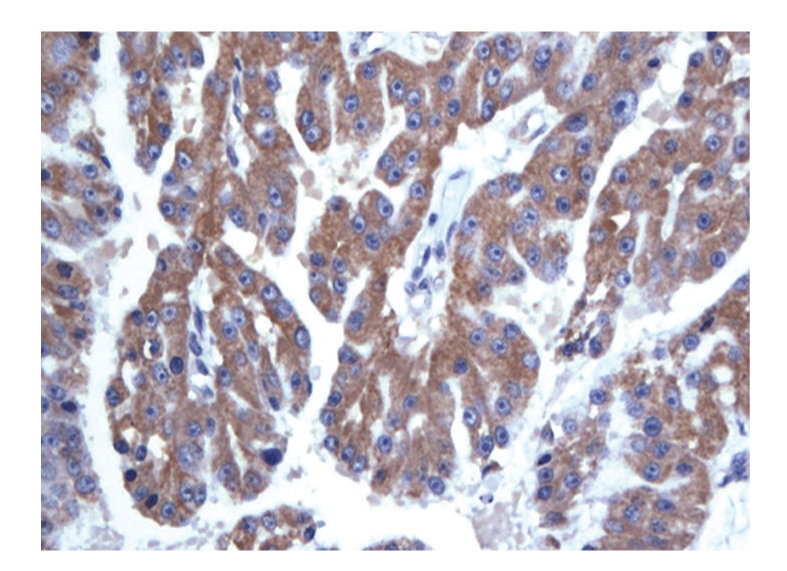
Immunohistochemical positive staining for trypsin marker.

A 48-year-old Caucasian male presented in September 2007 with a 10-year history of intermittent epigastric pain. He reported a recent increase in frequency of the pain, but not in severity. There were no other gastrointestinal symptoms such as weight loss, melena, hematochezia, nausea, vomiting, dysphagia, change in bowel habits, or jaundice. Serum amylase, lipase, and tumor markers were normal (CA 19-9: 7 KU/L, reference range: <35 KU/L; CEA: 1.0 *μ*g/L, reference rage: ≤5 *μ*g/L). A combination of imaging studies, including transabdominal ultrasound and computed tomography (CT) of the abdomen and pelvis (Figure 1), showed a large heterogeneous cystic lesion in the head of the pancreas measuring 10 × 6.9 × 5.5 cm displacing the second segment of the duodenum laterally. There were no loco regional lymph nodes, vascular invasion or distant metastases. The pancreatic duct in the body and tail was only mildly dilated (4 mm), and the biliary tree was not obstructed. Endoscopic ultrasound (EUS) of the pancreatic mass was performed utilizing a linear curved array echo endoscope (EG-3630U Pentax Medical Co, Montvale, NJ). A large heterogeneous cystic lesion with smooth margins measuring 10 × 7 cm was seen in the head of the pancreas. There were internal solid components adherent to the wall of the lesion but no septations. The pancreatic body and tail were normal in appearances. Fine needle aspiration of both the cystic and solid components of the lesion was performed using a 22 gauge; 8 cm needle (Echotip, Wilson-Cook Medical, Inc., Winston-Salem, NC) in 7 passes. Approximately 2 cc of thick viscous fluid was aspirated. Unfortunately, the aspirated cyst fluid was deemed unsuitable for cyst fluid analysis of CEA and amylase levels. Cytology showed malignant cells consistent with pancreatic carcinoma. The patient underwent pancreaticododudenectomy in November 2007 Pathological examination showed a large cystic lesion involving the head of the pancreas with papillary and nodular projections (Figure 2). The tumor was confined to the pancreas with no vascular invasion. No tumor was identified in 6 resected lymph nodes. Histological section showed a papillary tumor with fibrovascular cores covered by uniform cells with granular apical accentuation characteristic of acinar cell carcinoma (Figure 3). By immunohistochemistry, the tumor cells were positive for CAM 5.2, amylase, trypsin (Figure 4) and focally for synaptophysin, and negative for vimentin, insulin, glucagons, somastostatin, and electron microscopy confirmed the presence of zymogen granules characteristic of acinar cell carcinoma. The patient had chemotherapy postoperatively. He remains alive and well as of July 2009.

## 3. Discussion

Acinar cell carcinomas of the pancreas are rare tumors and account for less than 2% of all pancreatic carcinomas. The disease most often presents in the seventh decade of life, but can occur at any age. There is a 2 : 1 male predominance. The presenting symptoms are generally nonspecific including abdominal pain and weight loss. In contrast to ductal adenocarcinoma, jaundice is rare [1]. A minority of patients may develop a syndrome consisting of lipase hypersecretion characterized by subcutaneous fat necrosis, polyarthralgia, and eosinophilia [4]. The long-term survival for acinar cell carcinoma is poor, however several studies have confirmed that the clinical course is less aggressive than that of ductal adenocarcinoma [5]. Macroscopically, acinar cell carcinoma is typically a large solid and well-circumscribed tumor with rare cystic degeneration. The literature includes rare cases of small-sized intraductal and papillary variants of acinar cell carcinoma [3]. Microscopically, these tumors exhibit papillary-shaped epithelial projections with well-formed fibrovascular cores lined by cuboidal cells with acidophlic apical granules. Immunohistochemically, the tumor cells are positive for trypsin, lipase, and chymotrypsin, which are specific markers for acinar cell differentiation. By electron microscopy, these tumors exhibit characteristic zymogene granules. The current case is the first large 10 cm mass with a papillocystic pattern of acinar cell carcinoma. Recently, there have been advances in the classification of pancreatic neoplasia, including the recognition and better characterization of intraductal neoplasms. In addition to the microscopic dysplastic changes seen with ductal adenocarcinoma, now referred to as pancreatic intraepithelial neoplasia, there is also a group of mass forming intraductal neoplasm, in particular, intraductal papillary mucinous neoplasms. However in female patients mucinous cystic neoplasms and solid pseudopapillery tumors are to be included in the differential diagnosis [3]. All lesions exhibit intracystic papillary formation. Cystic neoplasms of the pancreas represent a diverse collection of tumors with varied malignant potential and clinical presentation. They can be predominantly cystic or can result from cystic degeneration of a solid tumor. Acinar cell carcinoma with papillocystic pattern can mimic these tumors, and attention to morphologic details, applications of immunohistochemistry and electron microscopy can be useful in establishing the accurate diagnosis. The follow-up on the few reported cases of papillocystic variant of acinar cell carcinoma in the literature showed less aggressive course than the traditional solid variant [3]. This variant may have different biological behavior, but the number of reported cases is too small to draw a definite conclusion. The conventional imaging methods that are used to evaluate cystic lesions of the pancreas are computed tomography (CT), magnetic resonance imaging (MRI), and endoscopic retrograde cholangiopancreatography (ERCP). High-resolution CT, using thin sections with both enhanced and unenhanced technique, provides detailed information about cyst structure and may facilitate characterization of these lesions [6]. MRI has the potential added advantage of determining communication between the cyst and pancreatic duct. ERCP is invasive but is more effective in visualizing the ductal anatomy. Despite the advancement in cross-sectional imaging technologies, radiographic differentiation of neoplastic from benign pancreatic cystic lesions remains difficult [7]. EUS on the other hand provides more detailed images of both pancreatic parenchyma and ductal anatomy at the same time, and also permits sampling of cyst fluid, mass lesions, and lymph nodes [7]. for pancreatic carcinoma, EUS appears to be superior to conventional imaging including CT, MRI, ERCP, and angiography, particularly for small masses less than 2-3 cm in diameter [8]. It has been reported in several studies that EUS has a sensitivity of over 95% for imaging pancreatic tumors 2 cm or less in diameter For pancreatic cystic lesions, a recent prospective, multicentre study of 112 cysts diagnosed by surgical resection or positive FNA found a cyst fluid CEA level of 192 ng/mL to be accurate in differentiating mucinous from nonmucinous pancreatic cysts (sensitivity 75% and specificity 84%) [7].

## 4. Conclusion

We have presented a rare case of papillocystic variant of acinar cell carcinoma. Acinar cell carcinoma rarely exhibit papillary or papillocystic pattern, and, therefore, fall into the challenging differential diagnosis of papillary and cystic pancreatic tumors. The correlation of clinical, radiographic, histological, and immunohistochemical findings would be helpful to distinguish such a tumor from other pancreatic papillary lesions as they carry different prognostic outcome.

## References

[1] Matos JM, Schmidt CM, Turrini O (2009). Pancreatic acinar cell carcinoma: a multi-institutional study. *Journal of Gastrointestinal Surgery*.

[2] Adsay N (2008). Cystic neoplasia of the pancreas: pathology and biology. *Journal of Gastrointestinal Surgery*.

[3] Basturk O, Zamboni G, Klimstra DS (2007). Intraductal and papillary variants of acinar cell carcinoma: a new addition to the challenging differential diagnosis of intraductal neoplasms. *The American Journal of Surgical Pathology*.

[4] Klimstra DS, Adasy NV, Odze RD (2004). Benign and malignant tumors of the pancreas. *Surgical Pathology of the GI Tract, Liver, Biliary Tract and Pancreas*.

[5] Schmidt CM, Matos JM, Bentrem DJ, Talamonti MS, Lillemoe KD, Bilimoria KY (2008). Acinar cell carcinoma of the pancreas in the United States: prognostic factors and comparison to ductal adenocarcinoma. *Journal of Gastrointestinal Surgery*.

[6] Morana G, Guarise A (2006). Cystic tumors of the pancreas. *Cancer Imaging*.

[7] Brugge WR, Lewandrowski K, Lee-Lewandrowski E (2004). Diagnosis of pancreatic cystic neoplasms: a report of the cooperative pancreatic cyst study. *Gastroenterology*.

[8] Bryne MF, Jewell PS (2002). Gastrointestinal imaging: endoscopic ultrasound. *Gastroenterology*.

